# Shear Mechanical Properties and Damage Deterioration of Anchored Sandstone–Concrete Under Freeze–Thaw Cycles

**DOI:** 10.3390/s26082458

**Published:** 2026-04-16

**Authors:** Taoying Liu, Qifan Zeng, Wenbin Cai, Ping Cao

**Affiliations:** School of Resources & Safety Engineering, Central South University, Changsha 410083, China

**Keywords:** sandstone–concrete, anchor blot, freeze–thaw cycles, straight shear test, acoustic emission, digital image

## Abstract

Acoustic emission (AE) and digital image correlation (DIC) techniques enable real-time capture of damage signals and full-field deformation at anchored rock–concrete interfaces under shear loading, which is critical for quantitatively characterizing freeze–thaw (F-T) degradation and preventing geological disasters in cold regions. This study synchronously monitored full-shear-process AE signals using a broadband AE system (150 kHz resonant frequency, 5 MS/s sampling) and captured high-precision full-field deformation via a 5-megapixel monocular DIC system (25 fps). F-T cycle and direct shear tests were conducted on sandstone–concrete anchored specimens with varying F-T cycles and anchor depths to investigate their effects on shear mechanical properties, AE characteristics and failure modes. Results show that AE peak ring count first decreases by 44.9% then increases by 56.5%, while cumulative ring count exhibits a three-stage evolution. Shear crack proportion first decreases then increases, with tensile failure remaining dominant throughout. DIC reveals that F-T cycles shift failure from crack propagation to surface delamination and interface slip, while different anchor depths induce distinct failure patterns. This study confirms that AE and DIC can accurately characterize F-T degradation, providing a reliable non-destructive monitoring method for cold-region anchorage engineering.

## 1. Introduction

In recent years, with the successive implementation of major national strategies such as the Belt and Road Initiative and the Western Development Strategy, China has accelerated the development and utilization of abundant resources in cold regions, leading to a gradual increase in construction projects in these areas [1]. Engineering structures in cold regions inevitably interact with geological formations. Tunnel construction, dam stability, and slope support require strong bonding between concrete and rock/soil [2,3]. However, the rock–concrete surface represents a weak point in these structures. Subjected to freeze–thaw cycles and external loads, it is prone to crack propagation and expansion [4], leading to debonding between the engineering structure and the underlying geology. Anchor technology is an effective reinforcement measure widely applied in rock mass and soil slope stabilization projects [5]. Therefore, an in-depth investigation into the effects of freeze–thaw cycles on the degradation of sandstone–concrete surfaces and the influence of different anchoring methods on the shear mechanical properties of sandstone–concrete is crucial for maintaining the safety and reliability of cold region protection projects.

Current research on freeze–thaw damage degradation in rock has yielded substantial findings. For instance, Lu Yanni and Li Xinping conducted freeze–thaw cycle and uniaxial compression tests on sandstone with varying cycle numbers, establishing relationships between rock strength parameters and bedding angle. They observed an increasing anisotropy in rock properties with rising freeze–thaw cycle counts [6]. Mutlutuk M et al. conducted freeze–thaw cycles on 10 rock types and concluded that repeated temperature fluctuations cause integrity loss, with higher cycle frequencies and greater temperature fluctuations leading to more severe integrity degradation [7]. Mu et al. conducted shear failure tests on three jointed rock specimens after varying freeze–thaw cycles, highlighting the critical role of freezing direction in fracture propagation. Using an ice wedge model, they also derived a theoretical solution for ice pressure within fractures [8].

As a relatively weak layer in engineering construction, the rock–concrete interface is more prone to debonding and cracking compared to the constituent materials of rock and concrete [9,10,11,12], leading to the failure of rock–concrete protection. Numerous scholars have studied the degradation of rock–concrete interfaces under loading and achieved valuable results [13,14,15,16]. V. Andjelkovic et al. [17] investigated the shear mechanical properties of rock–concrete under varying JRC values at the surface, establishing models for shear deformation and strength at the surface. Wei et al. [18] conducted freeze–thaw cycling and interfacial shear mechanics tests at varying shear rates on rock–concrete composites. They observed decreased interfacial shear strength with increasing freeze–thaw cycles and identified the shear damage mechanism as “abrasion, extrusion, and shearing” between particles.

A rock anchoring system is a collective system composed of multiple rock anchoring subsystems with the functional objective of achieving rock anchoring [19]. Research by Kilic et al. demonstrated that shear failure tests on rock bolts and grout materials confirmed the primary failure mode as shear failure of the grout material [20]. He et al. [21] investigated the shear mechanical behavior at the interface between soil and cement grout blocks under varying soil temperatures, moisture contents, and atmospheric pressure. Results indicate that the peak cohesive force at the interface increases with decreasing temperature, and increasing moisture content with this effect is more pronounced at lower temperatures. Cusson R et al. subjected GFRP reinforcement specimens to 250 freeze–thaw cycles and tested tensile strength, elastic modulus, ultimate strain, and shear strength. Results indicated that freeze–thaw cycles adversely affect GFRP reinforcement, with the extent of impact related to cycle number and temperature variation range. The strength reduction caused by freeze–thaw cycles did not exceed 10% [22]. Mansouri, Arezki, et al. improved the identification of localized damage in these types of joints by employing vibration-based and static load methods [23].

In summary, current research on the bond performance of rock–concrete interfaces under anchorage during freeze–thaw cycles remains limited. Studies primarily focus on the macro-mechanical degradation of interfaces, with few investigations into micro-scale phenomena such as pore expansion at the interface during freeze–thaw processes. This study uses direct shear tests to investigate the mechanical shear properties of a sandstone–concrete binary under anchorage and freeze–thaw cycles. Changes in freeze–thaw damage within the binary specimens were measured and analyzed using nuclear magnetic resonance (NMR) and wave velocity meters. The failure evolution during shear loading was monitored using acoustic emission (AE) and digital image correlation (DIC). This study designed and conducted direct shear tests on sandstone–concrete binarys under coupled conditions involving varying numbers of freeze–thaw cycles and anchorage depths. For the first time, this study systematically investigated the regulatory mechanisms of key anchoring parameters on the shear mechanical properties and anchoring performance of the binary system after freeze–thaw cycles. It fills a gap in systematic research on the performance of rock–concrete interfaces under coupled freeze–thaw and anchoring conditions. Real-time monitoring of the composite’s crack initiation, propagation, and penetration behavior was conducted throughout the entire freeze–thaw and shear loading process. The evolution patterns of interface shear failure under freeze–thaw conditions and for different anchoring parameters were clarified. This study provides direct experimental evidence and theoretical support for the long-term performance evaluation and parameter optimization of anchoring engineering in cold regions.

## 2. Sample Preparation and Test Methods

### 2.1. Sample Preparation

**Figure 1:**
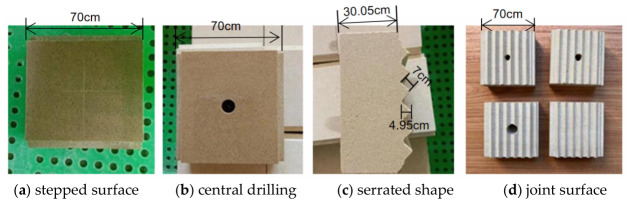
Specimen processing procedure.

**Figure 2:**
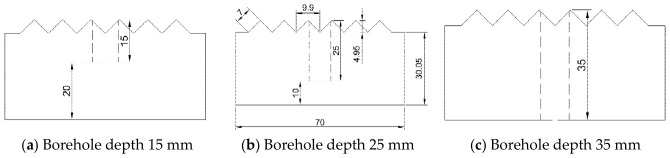
Sample size.

There are four anchorage depth variables: 0 mm, 15 mm, 25 mm, and 35 mm. There are five variables for the number of freeze–thaw cycles: 0, 10, 20, 30, and 40. These two sets of variables are combined in an orthogonal arrangement. Three groups are established for each specimen type, for a total of 60 specimens. All data used in the following article are averages calculated after excluding outliers. This test selected fine-grained sandstone with uniform texture and no distinct bedding. Its primary mineral components consist of quartz (SiO_2_, volume fraction 38–65%) and feldspar (NaAlSi_3_O_8_-KAlSiO_8_, volume fraction 22–41%), with minor constituents including mica (volume fraction 5–9%), clay minerals (3–7% by volume), and iron oxides (1–4% by volume). Homogenization treatment redistributed internal stresses within the sandstone, reducing structural variations. Rock samples were cut into 70 mm × 70 mm × 35 mm rectangular prisms, and Φ50 mm × 100 mm cylindrical cores were extracted [24]. Subsequently, serrated cuts were made in the 70 mm × 70 mm cross-section. Detailed processing steps are shown in Figure 1, and the specimen dimensions are illustrated in Figure 2.

**Figure 3:**
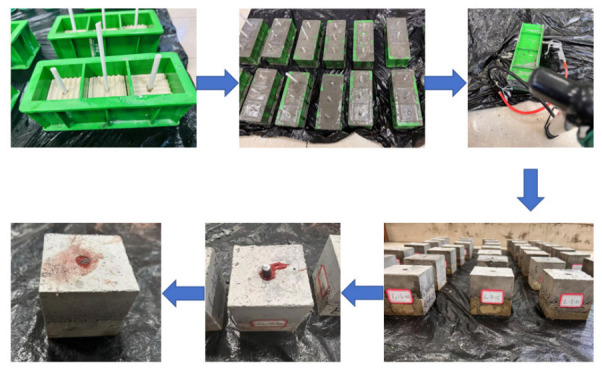
Process for preparing anchor sandstone–concrete binary specimen.

Anchor bolts were simulated using 6 mm-diameter straight threaded bolts. The anchor bolts were available in three lengths: 15 mm, 25 mm, and 35 mm. Modified epoxy resin was employed as the anchoring agent. Ordinary Portland cement (P·O42.5 grade), purified water, sand, and gravel were mixed in a container at a mass ratio of 1:0.56:2.06:3.65 and stirred for 90 s. Release agent was applied evenly to PVC tubes with an 8 mm outer diameter and were then inserted into mold boxes. The mixed concrete was poured into the mold boxes and vibrated for 20–30 s using a vibrating table. The PVC tubes were removed, allowing the specimens to rest at room temperature for 24 h, then demolded. The specimens were cured for 28 days at 20 ± 2 °C and ≥95% relative humidity. After injecting epoxy resin into the anchor holes, the bolts were inserted. They were allowed to stand for 24 h to complete the preparation of the sandstone–concrete composite specimens with bolts. The primary fabrication process is illustrated in Figure 3:

### 2.2. Freeze–Thaw Cycling Test and Mechanical Test

**Figure 4:**
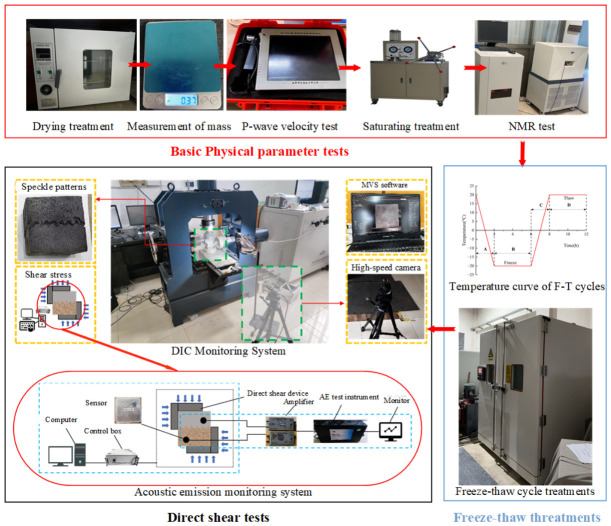
Experimental procedures and equipment.

Figure 4 presents the experimental devices and procedures in this study.

The specimens were placed in an electric heating constant-temperature drying oven (Model 101-1A, SHAHNGCHENG INSTRUMENT, Shaoxing, China) for drying at 105 °C for 24 h. After cooling, the mass was determined using an electronic balance. Subsequently, the specimen’s P-wave velocity was measured using a rock wave velocity tester (Model HS-YS4A, Xiangtan Tianhong Electronics Co., Ltd, Xiangtan, China). The specimen was then saturated using a vacuum pressure device (Model ZYB-II, UNIPAC TECHNOLOGY, Haian, China), with a 4 h evacuation period and a 2 h dehumidification period. Afterwards, the specimen was immersed in distilled water for 48 h to complete saturation, followed by reweighing.

Building upon the findings of Guo et al. [25], the present work is designed to replicate the actual engineering conditions encountered in cold regions. The saturated specimen was placed in a high- and low-temperature test chamber. First, the temperature was lowered to −20 °C for 2 h, then frozen for 4 h. Then, the temperature was raised 20 °C for 2 h, followed by thawing for 4 h. One freeze–thaw cycle was completed. Samples were divided into five groups, each subjected to 0, 10, 20, 30, or 40 freeze–thaw cycles. After completing the freeze–thaw cycles, the samples were saturated, dried, weighed, measured for length, and retested for P-wave velocity.

For nuclear magnetic resonance (NMR) testing, cylindrical specimens (Φ50 mm × 100 mm) were used to determine porosity and to measure T2 spectrum distributions via NMR for sandstone and concrete specimens subjected to varying freeze–thaw cycle counts.

**Figure 5:**
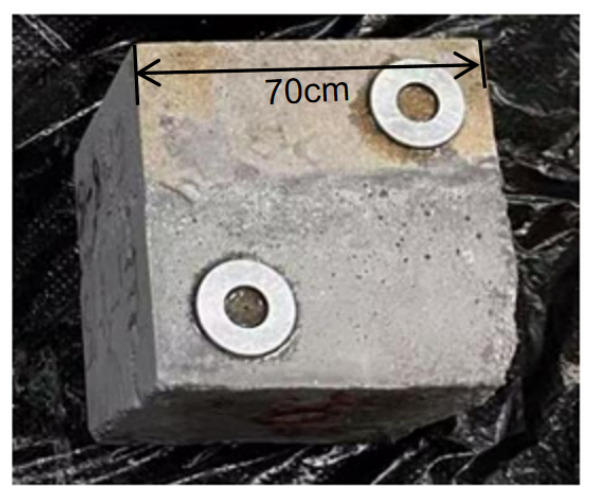
Bond spacers on binary specimen.

After freeze–thaw cycles, specimens undergo uniaxial shear mechanical testing to investigate changes in rock mechanical properties, surface crack propagation, and acoustic emission characteristics under three variables: freeze–thaw cycles, bolt length, and bolt depth. The specific procedure is as follows:

(1) DIC Monitoring System: Before the direct shear test, speckle patterns were sprayed onto the specimen surface. An industrial high-speed camera was positioned directly facing the specimen, and image calibration was performed using MVS_STD (v4.4.1, Hangzhou Hikrobot Co., LTD, Hangzhou, China) software.

(2) Acoustic Emission Monitoring System: Dual-channel acoustic emission signal monitoring was employed during the direct shear process. First, two spacers were bonded as shown in Figure 5. The probe was secured to the spacers using magnetic caps, connected to a preamplifier (internal gain 40 dB), and then linked to the acoustic emission acquisition and analysis device.

(3) Direct Shear Test: First, a preload is applied to the specimen in the normal direction, loading up to a target value of 0.5 kN. Subsequently, a normal force is applied at a rate of 0.5 kN/s will until a target value of 9.8 kN is reached. At this point, the specimen’s normal stress is 2 MPa. Next, a tangential load is applied using displacement control at a rate of 0.01 mm/s, with a maximum displacement of 50 mm. After specimen failure, testing ceases once the shear stress stabilizes, and then the DIC and acoustic emission equipment are deactivated.

## 3. Result Analysis

### 3.1. Physical Characteristic Analysis

**Figure 6:**
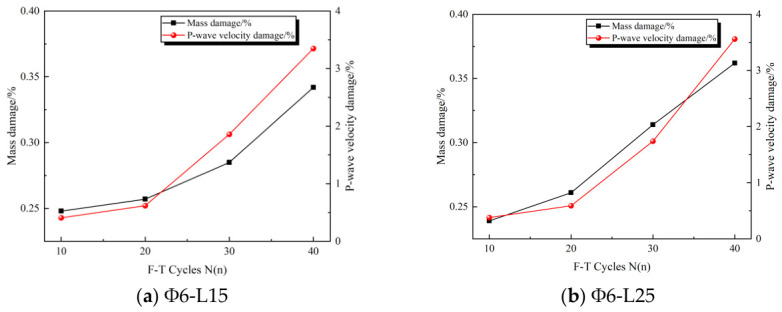

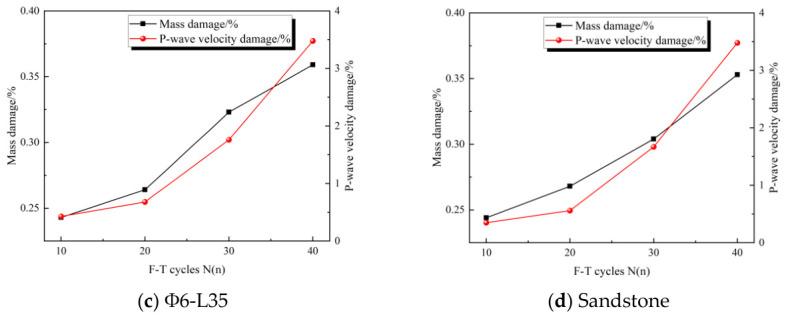
Changes in mass and P-wave velocity damage of specimens of different sizes under different freeze–thaw cycle counts.

Figure 6 illustrates the variations in the specimen mass and P-wave velocity increasing F-T cycles. During the initial stage of the F-T cycles, the specimens exhibit minor loss and P-wave velocity reduction. However, as the number of F-T cycles increases, the extent of mass loss becomes progressively more pronounced. Over 20–40 F-T cycles, the average mass loss of the specimens increases from 0.26% to 0.35%, representing a 34.62% increase. Concurrently, the average P-wave velocity reduction rises from 0.63% to 3.5%, corresponding to a 455.56% increase. The magnitude of P-wave velocity reduction is approximately 13 times that of mass loss over this interval, indicating that P-wave velocity is far more sensitive to F-T cycles than mass. The underlying mechanism can be explained as follows. During the freezing phase, the internal cracks in the specimens expend under the action of frost heaving forces, and the specimens transition from a saturated to an unsaturated state. Detached particles generated by crack propagation are temporarily trapped within the pore structure. During the subsequent thawing phase, ice crystals in the pores melt rapidly, creating negative pressure (suction) within the specimens. This negative pressure drives water reabsorption, which flushes out the trapped detached particles, leading to further mass loss and P-wave velocity reduction. In the later stages of F-T cycles, owing to the inherent heterogeneity of rock-forming particles, repeated F-T cycles progressively weaken inter-particle bonds. This accelerates the initiation and propagation of micro-fractures, and the continuous accumulation of F-T damage further exacerbates mass loss and P-wave velocity reduction.

### 3.2. Nuclear Magnetic Resonance Analysis of Rocks and Concrete Under Freeze–Thaw Cycling

#### 3.2.1. T2 Spectrum Analysis

**Figure 7:**
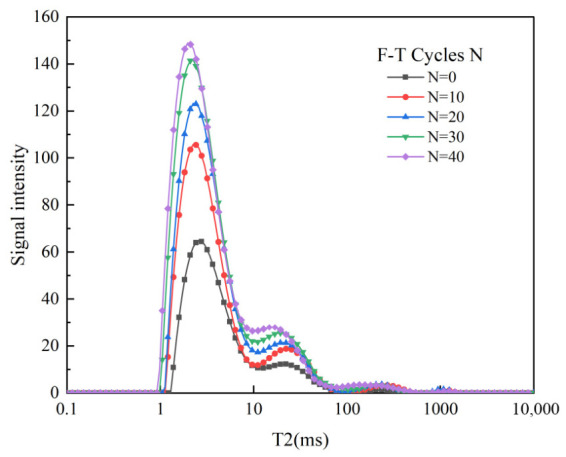
Distribution of transverse relaxation time T_2_ in sandstone under different F-T cycle counts.

The T2 spectrum distribution curve reflects changes in the rock’s pore structure. The magnitude of the horizontal coordinate is positively correlated with the pore size. The signal on the vertical axis represents the relative number of pores [26,27,28]. As shown in Figure 7, the T2 spectrum of sandstone exhibits four peaks. From left to right, these correspond to small pores, medium pores, large pores, and micro-fractures. The signal intensity decreases sequentially, indicating the abundance of pores within the sandstone: small pores > medium pores > large pores > micro-fractures. During the early stage of freeze–thaw cycles, the signal amplitude of Peak 1 increases significantly In later stages, the amplitude continues to rise, but the rate of increase is gradually slowed, while the relaxation times corresponding to each peak remain largely unchanged. The signal amplitude of Peak 2 shows a similar trend. The relaxation times at the peak maxima show slight variation. The signal amplitude of Peak 3 follows a pattern of initial increase, followed by a decrease, and then a slight rise. The relaxation time of Peak 3 varies significantly between 10 and 40 F-T cycles: it increases notably from 0 to 10 cycles and then decreases markedly from 30 to 40 cycles. Peak 4 is absent in unfrozen sandstone. As F-T cycles progress, the signal amplitude of Peak 4 gradually increases but experiences a sharp decline at 40 cycles. At this stage, the relaxation time for Peak 4 is longer than during the first 30 cycles. The relaxation times show only slight variation during the first 30 F-T cycles.

The analysis indicates that small and medium pores become more prevalent as the number of F-T cycles increases. The number of small and medium pores continues to grow, while that of large pores increases slightly. Consequently, the signal amplitudes Peaks 1 and 2 increase with the number of F-T cycles. The emergence of Peak 4 with a minimal amplitude suggests that some large pores gradually develop into micro-fractures during the later stages of freeze–thaw cycles. The peaks corresponding to large pores shift to shorter relaxation times, whereas those for micro-fractures shift to longer relaxation times. These changes are accompanied by minor surface spalling of the sandstone, resulting in partial loss of fracture signals. Hence, the signal amplitudes of Peaks 3 and 4 decreased during the later stages of F-T cycles.

#### 3.2.2. T2 Spectrum Area and Pore Volume Quantification Analysis

**Figure 8:**
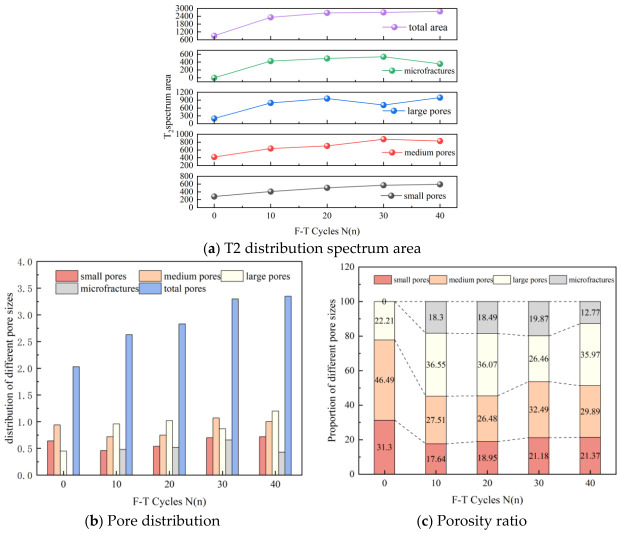
T2 distribution spectrum area, pore distribution and porosity ratio of sandstone.

The relaxation time is divided into four levels: small pores (0–10 ms), medium pores (10–100 ms), large pores (100–1000 ms), and micro-fractures (1000 ms and above) [29,30,31]. The distribution of pore sizes under different F-T cycles is determined by sequentially partitioning and integrating the T2 spectrum based on pore size. The distribution and proportion of different pore sizes in sandstone are statistically analyzed, resulting in Figure 8. As shown in Figure 8a, the total area of the T2 spectrum in sandstone continues to increase with the number of F-T cycles, indicating that F-T cycles significantly increase the number of pores in sandstone. The T2 spectrum area curve for small pores gradually increases with the number of F-T cycles. The T2 spectrum area curve for medium pores increases from 0 to 30 F-T cycles, then decreases. The T2 spectrum area curve for large pores increases before 30 F-T cycles and then decreases and rebounds. The T2 spectrum area curves for micro-fractures and medium pores converge, both decreasing after 30 F-T cycles. Since the T2 spectrum area reflects pore content, the number of small pores increases with the number of F-T cycles. Conversely, the numbers of medium pores, large pores, and micro-fractures all exhibit reduction phases at different F-T cycles. Based on the analysis of Figure 8b,c, during the first 0–10 F-T cycles, the proportions of small and medium pores decreased, corresponding to a decrease in porosity. The porosity of small pores decreased from 0.64% to 0.46%, and the porosity of medium pores decreased from 0.94% to 0.72%. While the proportions of large pores and micro-fractures increased, porosity increased. The porosity of large pores increased from 0.45% to 0.96%, and that of micro-fractures increased from 0% to 0.48%. The T2 spectrum area of all pore types increased, indicating that F-T cycles increased the number of each pore type. Sandstone underwent F-T cycles, forming new small pores. Previous small-to-large pores evolved into medium pores and micro-fractures, primarily through the evolution of medium pores into large pores and large pores into micro-fractures. These resulted in an increase in the proportion of large pores and microfractures, while the proportions of other pore types decreased. After 10–20 F-T cycles, the proportions of small pores and microfractures increased, leading to a corresponding increase in porosity. The proportions of medium pores and large pores decreased slightly. However, their corresponding porosities increased. Medium-pore porosity rose from 0.72% to 0.75%. Moreover, the proportion of large pores increased from 0.96% to 1.02%. This is because the accumulation of F-T damage accelerates pore expansion. Sandstone has a high total porosity. Even if the proportion of pores decreases, the corresponding porosity remains relatively high when multiplied by the total porosity. At this stage, the main processes are the formation of small pores and the expansion of micro-fractures. After 20–30 F-T cycles, the overall porosity significantly increased from 2.83% to 3.3%. The content of large pores decreased, with the proportion dropping from 36.07% to 26.46%. Moreover, the corresponding porosity decreased to 0.87%. The proportions of other pore types all increased significantly. This is because during this stage, large pores develop into microfractures more rapidly. A large number of large pores evolve into microfractures, while other pore types primarily form small pores that expand into medium pores. After 30–40 F-T cycles, the process enters the late F-T stage. The total porosity continues to increase. The proportions and porosity of small and large pores both increase. In contrast, the proportions and porosity of medium pores and micro-fractures both decreased, and the content of microfractures also decreased. The analysis suggests that the cause may be surface spalling of the sandstone due to long-term F-T cycles. Mass loss leads to the disappearance of microfracture signals, thereby reducing the measured T2 spectrum area.

### 3.3. Shear Stress–Shear Displacement Curve

**Figure 9:**
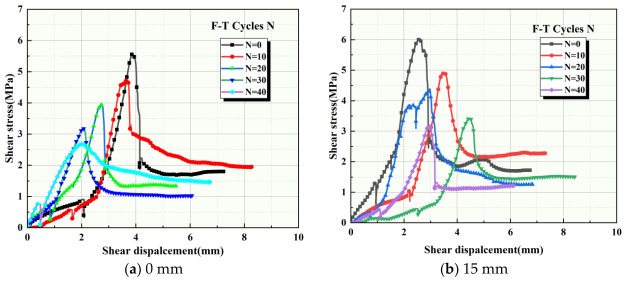

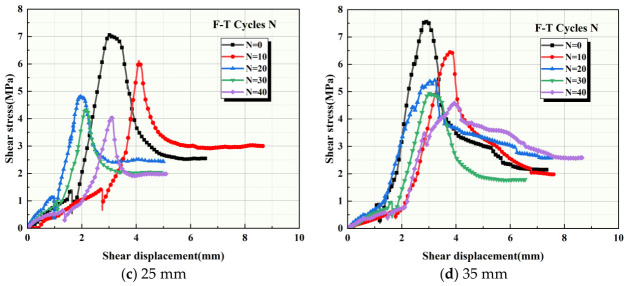
Shear stress–displacement curves of binary specimens under different freeze–thaw cycle numbers.

In this test, the normal stress is kept constant. The anchorage depths of the samples in the same group are the same, with a uniform bolt diameter of 6 mm. The samples are divided into four groups based on anchorage depth: 0 mm, 15 mm, 25 mm, and 35 mm. After completing the direct shear test, the data were obtained from the rock direct shear tester control system. The shear stress–shear displacement curves of the binary material specimens in each group are shown in Figure 9.

As shown in Figure 9, under different F-T cycle counts, the shear stress–displacement curves of specimens with different anchorage depths in each group exhibit similar trends: (1) The peak shear strength of specimens in each group is highest at 0 F-T cycles. As the number of F-T cycles increases, the peak shear strength decreases to varying degrees, with the minimum value occurring at 40 F-T cycles. (2) Compared to the unanchored specimens, the peak strength of the anchored specimens is significantly higher and increases with increasing anchorage depth. This indicates that anchorage depth strengthens peak shear strength, and the strengthening effect of anchor bolts on peak shear strength is significant under 0 and 40 F-T cycles. (3) In all groups, shear stress continuously increases with increasing shear displacement during the shear process, reaching a peak shear stress, decreasing, and finally stabilizing at a specific value, i.e., the residual shear strength. The residual shear strength for binary specimens within the same group also changes with different F-T cycles. The residual shear strength of unanchored binary specimens is similar at 0 and 10 F-T cycles, at 1.8 MPa and 1.95 MPa, respectively. However, as F-T cycles continue, the residual shear strength decreases significantly. The residual shear strength of other anchored specimens exhibits complex variations, but overall, the residual shear strength after 40 F-T cycles remains the lowest. (4) Compared to unanchored specimens, under the same number of F-T cycles, the residual shear strength of anchored binary specimens is relatively higher due to the bolt material’s higher strength than the rock itself. (5) Some specimens’ shear stress–shear displacement curves exhibit a double-peak shape, such as the curve for 30 F-T cycles in Figure 9b. This is due to the presence of sawtooth-like structures with high shear strength on the joint surfaces of the binary specimens. When these sawtooth structures on the joint surfaces fracture under tangential loading while the remaining ones remain intact, the shear stress–shear displacement curve may exhibit a double-peak or even multi-peak morphology.

**Figure 10:**
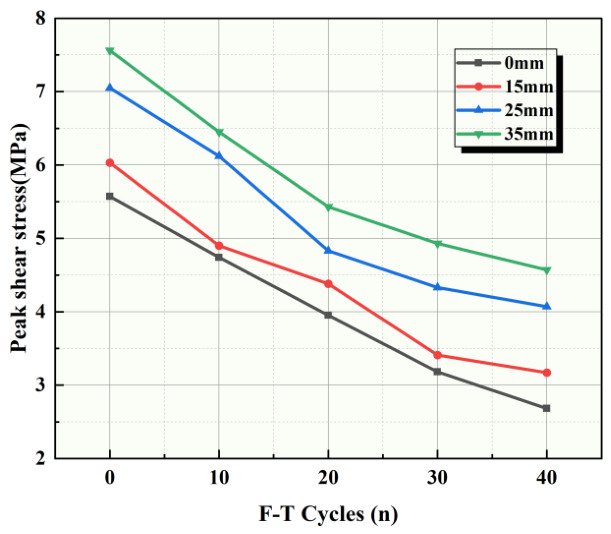
Peak shear strength versus freeze–thaw cycle count curve.

The peak shear strengths of each group under different F-T cycles were organized, and the relationship between peak shear strength and F-T cycles is shown in Figure 10. Peak shear strength decreases with increasing F-T cycles. For sandstone–concrete specimens with anchor bolts, due to the anchoring effect, the trend in peak shear strength differs from that of specimens without anchor bolts. The peak shear strength of specimens without anchor bolts decreases linearly with F-T cycles. For other specimens with anchor bolts, the peak shear strength decreases significantly during the initial F-T period of 0–20 cycles, after which the decrease slows.

### 3.4. Shear Stress–Shear Displacement Curves of Sandstone–Concrete Binary Specimens at Different Anchorage Depths

**Figure 11:**
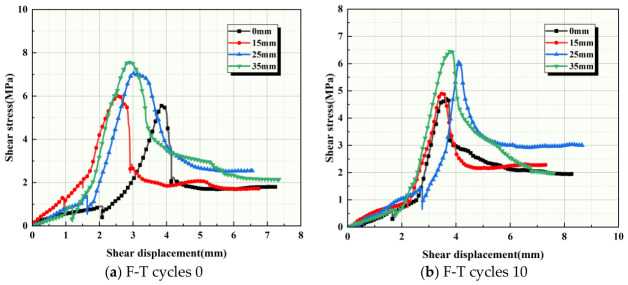

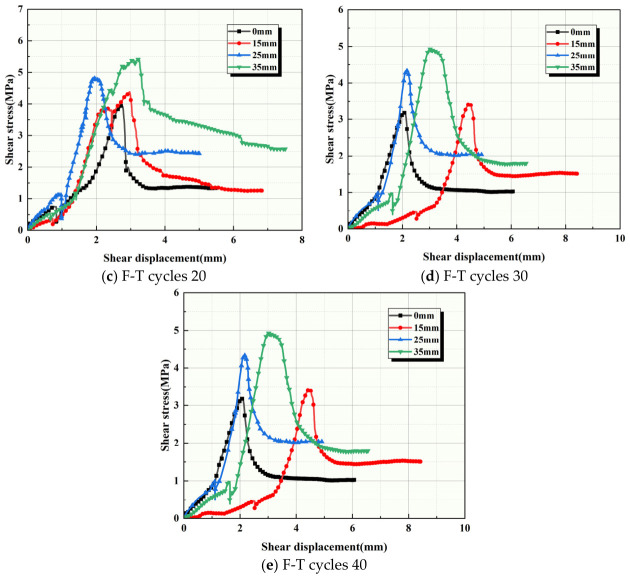
Shear stress–displacement relationship curves of binary specimens at different anchor bolt depths.

By organizing existing data, we analyzed the effect of anchorage depth on the shear strength of composite specimens subjected to the same number of F-T cycles. The trend diagrams of peak shear strength changes for composite specimens at different anchorage depths are shown in Figure 11.

(1) Comparing the shear strength–displacement changes of the binary specimens under the same F-T cycles, it can be observed that the peak shear strength is highest at an anchorage depth of 35 mm. As the anchorage depth decreases until there is no bolt, the peak shear strength of the binary specimens decreases to varying degrees. The peak shear strength under no anchors was the lowest among all groups. (2) Compared to specimens with 0 F-T cycles, the enhancing effect of anchor bolts on the shear strength of binary specimens is more pronounced at higher F-T cycles. This enhancing effect increases with increasing anchorage depth. (3) When comparing the changes in residual shear strength at different anchorage depths under the same F-T cycles, using F-T cycles of 0 as an example, it was found that the residual shear strength of the unanchored binary specimen was lower than that of the anchored specimen. The residual shear strength of the unanchored specimen with 0 F-T cycles is 1.7 MPa, while the residual shear strength at an anchorage depth of 15 mm is 1.73 MPa. The residual shear strength difference between 0 and 15 mm is small. However, as the anchorage depth increases, the residual shear strength changes significantly. Interestingly, the residual shear strength at an anchorage depth of 25 mm is higher than that at 35 mm. This is because, at greater anchorage depths, the bolt exceeds its critical length, leading to an uneven shear stress distribution and local stress concentration. The high degree of rock fragmentation reduces residual strength. Except for the 0 F-T cycle case, the binary specimens exhibit a similar trend after 10 F-T cycles. However, the binary specimens after 20 and 30 F-T cycles show different trends. The residual shear strengths ranked by anchorage depth as d0 ≤ d15 < d25 < d35, indicating a significant enhancement in anchorage effectiveness. F-T cycles not only degrade the rock but also weaken the bond strength and friction coefficient at the anchor–rock interface. At an anchorage depth of 15 mm, delamination or premature slippage occurs at the interface, compromising anchoring performance. At greater anchoring depths, although some joint surface degradation occurs, the longer anchoring segment provides a larger total bonding area and greater mechanical interlock. This allows for maintaining high residual shear strength even in the later stages of F-T cycles. This coupling effect between joint surface damage and anchoring length results in variations in the anchoring depth–strength relationship across different F-T stages. The residual shear strength is significantly improved at an anchorage depth of 35 mm. The analysis indicates that increased F-T cycles induce more extensive crack propagation under F-T action. This increases the effective friction area between the anchor bolts and the rock mass/joint surfaces, thereby elevating residual shear stress. After 40 F-T cycles, the specimens exhibited significant damage. At low anchorage depths, the bond strength of the anchor bolts decreased. The anchor bolts slipped, forming a weak zone. At this point, the residual shear strength became lower than that without anchor bolts.

**Figure 12:**
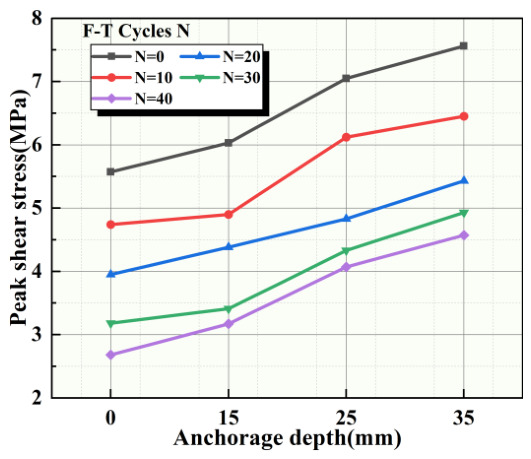
Relationship between peak shear strength and anchorage depth.

We organized the peak shear strengths of the binary specimens with different anchorage depths across all groups and obtained the relationship between the peak shear strengths and anchorage depths as shown in Figure 12. It was found that, for the same number of F-T cycles, the peak shear strength of the binary material generally exhibits a nonlinear increase trend with increasing anchorage depth. Under different F-T cycle counts, the peak shear strength of the binary material subjected to 40 F-T cycles is more sensitive to changes in anchorage depth. Increasing the anchorage depth from 15 mm to 25 mm has the greatest effect on peak shear strength. This change was most pronounced during the initial stages of F-T cycling.

### 3.5. Analysis of AE Results

#### 3.5.1. Characteristics of AE Counts

**Figure 13:**
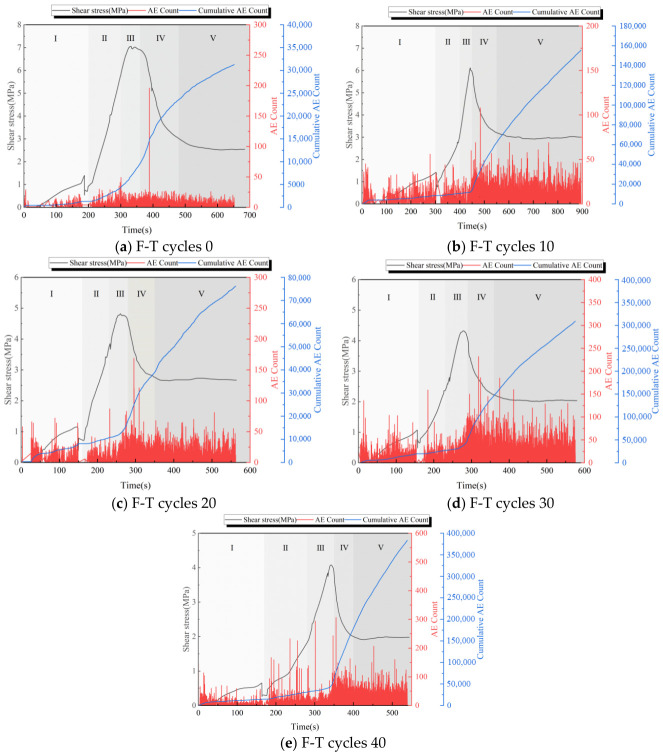
Characteristics of AE ringing counts and cumulative AE counts in binary specimen under different F-T cycles.

This section analyzes the acoustic emission count characteristics of sandstone–concrete binary specimens subjected to 0, 10, 20, 30, and 40 F-T cycles, using specimens with an anchorage depth of 25 mm and a bolt diameter of 6 mm as examples. Figure 13 illustrates the changes in shear stress, acoustic emission ringing counts, and cumulative acoustic emission ringing counts throughout the shear failure process of the specimens. Based on the shear stress–time curve characteristics of the composite specimens, five stages are identified: Stage I represents the initial compaction stage. Under shear loading, the primary internal fractures of the specimen undergo compaction. The AE ringing count exhibits a sharp increase over a short period, followed by weak fluctuations. Stage II is the elastic stage, where the specimen undergoes elastic deformation with minimal damage. The AE ring count fluctuates less than in the initial compaction phase, while the cumulative AE ring count increases linearly. Stage III is the yield stage. At this point, cracks initiate and propagate stably within the bimetallic specimen, gradually penetrating the material. Once the specimen reaches its peak shear strength, crack propagation becomes unstable and accelerates. During this stage, the AE ringing count gradually increases, and the increase in the cumulative AE ringing count accelerates. Stage IV is the failure stage. Crack propagation becomes unstable, deepening cracks appear, and numerous macroscopic cracks emerge on the surface. At this point, the AE ring count increases explosively, and the cumulative AE ring count rises rapidly. Stage V is the residual stage. The bimetallic specimen fails completely under shear stress, abruptly reducing its load-bearing capacity. However, it retains some shear load-bearing capacity due to joint friction and bolt effects. During this stage, the AE ringing count fluctuates stably, and the growth rate of the cumulative AE ringing count gradually flattens. Observing Figure 13a–e, as the number of F-T cycles increases, the AE ringing counts during the initial application of shear stress also rise. This is primarily due to F-T cycles, reducing the bonding strength between sandstone and concrete particles. The specimen developed additional primary cracks, or primary cracks that had not fully propagated during initial loading, which were triggered. This increased the initial acoustic emission ringing signal. The peak AE ringing count decreased from 196 to 108 between 0 and 10 F-T cycles. Subsequently, as F-T cycles increased, the peak count gradually rose. Meanwhile, the cumulative AE ringing count surged sharply from 0 to 10 cycles, dropped significantly at 20 cycles, and increased with further F-T cycles. This occurs because, between 0 and 10 F-T cycles, micro-cracks form between the bimetallic body and the bolt contact surface, and the interfacial bonding strength decreases. This reduces the cohesive force between the bolt and the rock mass and between the rock mass and the joint surface, making them prone to slippage under shear stress. Consequently, energy that should be concentrated and released is dispersed, reducing the intensity of individual AE events. Simultaneously, the bolt’s constraint effect on the composite prolongs the time to damage accumulation time, generating numerous low-energy AE events. This reduces the peak AE ringing count while increasing the cumulative AE ringing count. After 10 to 20 F-T cycles, the F-T action increases the porosity of the composite structure. Primary cracks further propagate and gradually connect to form macroscopic weak planes. Under shear loading, cracks propagate more readily along these weak planes. Shear energy is released primarily through major cracks. However, the number of events decreases, leading to an increase in AE ringing counts and a temporary decrease in cumulative AE ringing counts. During the later F-T cycles (20 to 40 cycles), F-T damage accumulates. The destruction of internal residual cement generates new cracks, thereby increasing the number of AE ringing events. Increased friction among rock fragments significantly increases low-energy events, resulting in a sustained increase in the cumulative AE ringing count.

#### 3.5.2. Analysis of Acoustic Emission RA-AF Feature

Tensile cracks and shear cracks are two types of fractures that appear during the loading failure process of rock specimens. When tensile failure dominates, the rock develops more tensile cracks, as evidenced by RA values that are lower than AF values. The RA-AF (RA = rise time/amplitude, AF = ring counts/duration time) distribution characteristics appear in the upper left and lower right regions, corresponding to the tensile and shear cracks, respectively. When shear loading predominates, shear cracks primarily form, indicated by RA values less than AF values. Through acoustic emission RA-AF analysis, the failure characteristics of rock under loading can be determined, and internal damage during rock specimen loading can be detected [32,33,34]. Dong et al. [35] conducted an expansion rupturing experiment and elastic wave attenuation experiments to investigate the uncertainties of the RA-AF method. And they use moment tensor results as the criterion to determine the reference line for micro-fracture-type division in the RA-AF distribution. The slopes of the reference lines for dividing the micro-fracture types are 0.59. Using the same criteria, this paper determines that the slope k is 0.59.

**Figure 14:**
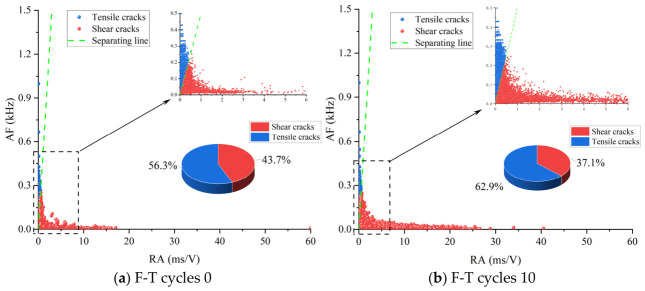

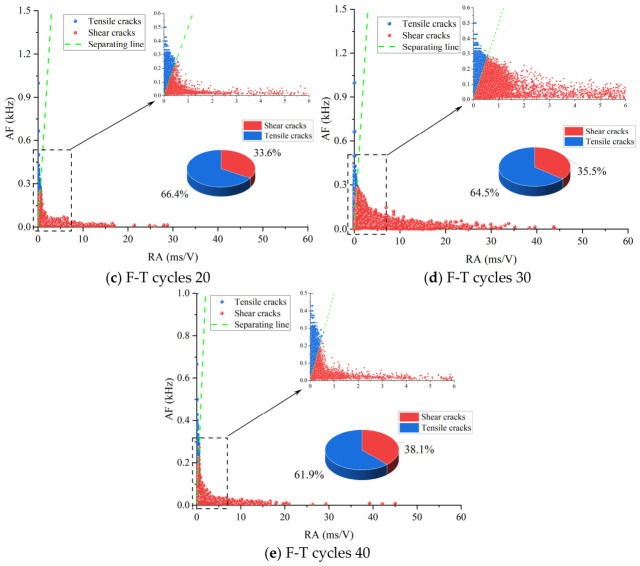
Classification of cracks in specimens after different freeze–thaw cycles.

In this section, sandstone–concrete binary specimens with a bolt depth of 25 mm and a bolt diameter of 6 mm were selected for AE RA-AF feature analysis. This study investigated the micro-crack characteristics of the composite specimens under direct shear loading after anchorage under different F-T cycle conditions. The RA-AF point density map is shown in Figure 14. As shown in Figure 14, the distribution of shear and tensile signals is generally similar across different F-T cycle counts. Most AF values are low while RA values are high. As the number of F-T cycles increases, more RA values with higher magnitudes appear in the AE signals, while the fluctuation range of AF values is not significant. The direct shear failure of the sandstone–concrete binary system is primarily dominated by tensile failure. For specimens that have not undergone F-T cycles, the extent of tensile failure is similar to that of shear failure, exhibiting a composite tensile–shear failure mode. As the number of F-T cycles increases, micro-cracks form at the rock–concrete interface and gradually propagate. The bond strength between sandstone and concrete decreases slightly, leading to debonding under shear loads and cracks propagating in the normal direction. Simultaneously, under the anchoring effect of anchor bolts, shear slip is constrained, forcing energy to be released through tensile cracks and suppressing crack propagation. In the later stages of F-T cycles, the debonding range between bolts and the surface expands, and the bolts’ constraint on shear slip decreases, shifting from active constraint to passive friction. The resistance to shear slip decreases, and accumulated F-T damage is created through cracks, forming dominant shear paths. The friction and slip between loose particles detached due to F-T contribute to the formation of additional shear cracks, increasing the proportion of shear cracks.

#### 3.5.3. Analysis of Acoustic Emission B-Values

**Figure 15:**
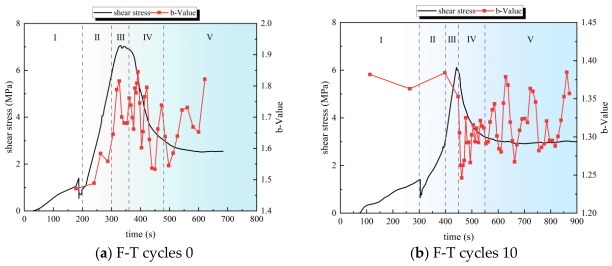

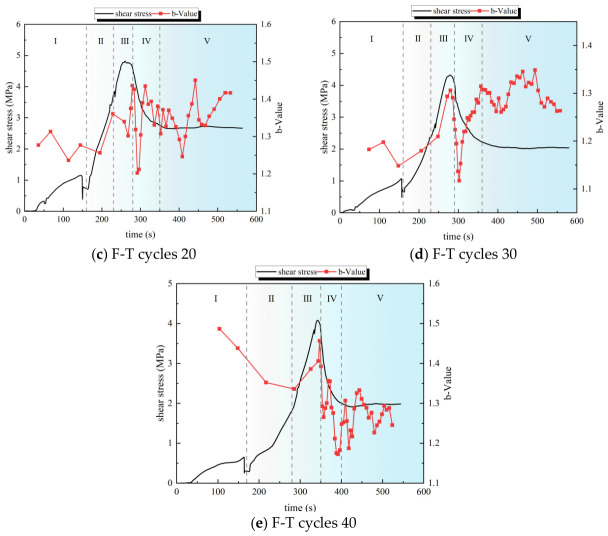
b-value curves of binary specimens under different freeze–thaw cycle numbers.

Based on the AE sampling frequency, 500 samples were collected, and 100 data points were selected using a sliding window. The B-value was calculated using Python 3.8. The B-value curves for the sandstone–concrete binaries under different freeze–thaw cycles were obtained, as shown in Figure 15.

As shown in Figure 15, the b-value of all specimens subjected to F-T cycles decreases significantly during the initial compaction stage (Stage I). In contrast, no such decrease in b-value is detected for the specimens without F-T treatment. F-T action weakens the inter-particle cementation force within the specimens, inducing particle dislocation and microcrack initiation at the initial compaction stage, thereby leading to a decrease in the b-value during this period. In contrast, the primary fissures in specimens without F-T treatment are compacted, resulting in a slight increase in the b-value. During the elastic stage (Stage II), the b-value of specimens with 0 to 30 F-T cycles shows an upward trend with fluctuations, among which the specimen with 0 F-T cycles has the largest increment amplitude of b-value. Conversely, the b-value of the specimen subjected to 40 F-T cycles presents a gradual decreasing trend, which is closely related to the increased amount of microcracks generated by cumulative F-T damage. As the shearing process progresses, the b-value of all specimens drops sharply at the end of the yield stage (Stage III) when it reaches or approaches its peak. This phenomenon indicates that macro-crack propagation occurs in the specimens, accompanied by severe damage. After entering the ductile failure stage (Stage IV), the b-value exhibits high-frequency, large-amplitude fluctuations, indicating that the damage of the specimens is further aggravated, with surface spalling. Specimens with low F-T cycles show frequent and large-amplitude fluctuations of b-value, while those with high F-T cycles exhibit a lower fluctuation degree of the b-value. The authors hold that F-T action degrades the bonding performance between the rock bolt and joint surfaces, as well as between adjacent joint surfaces. The failure of specimens with high F-T cycles is dominated by surface particle spalling, interface friction, and gnawing failure, which is distinct from specimens with low F-T cycles, where the inter-particle cementation remains favorable, and crack propagation is still prone to occur. During the residual stage (Stage V), the b-value of specimens with no more than 30 F-T cycles fluctuates significantly. The fluctuation frequency of the b-value for the specimen with 20 F-T cycles is lower than that with 10 and 0 F-T cycles, and the fluctuation frequency of the specimen with 10 F-T cycles is higher than that with 0 F-T cycles. This may be attributed to the specimens’ tendency to crack along the rock bolt during the late F-T stage, resulting in slight mutual extrusion between the rock bolt and the specimen.

In summary, for specimens with low F-T cycles, with the progression of shearing, the b-value of the binary specimen fluctuates and rises during and before the elastic stage. The b-value reaches or approaches its peak at the ductile failure stage, drops sharply near the final failure stage, and fluctuates violently in the residual stage. With an increase in F-T cycles, fluctuations in b-value during the residual stage are mitigated, and the AE events of the specimens are dominated by interfacial particle friction.

## 4. Analysis of Failure Modes

### 4.1. Effect of Freeze–Thaw Cycles on the Shear Failure Mode of Sandstone–Concrete Binary Specimen

**Figure 16:**
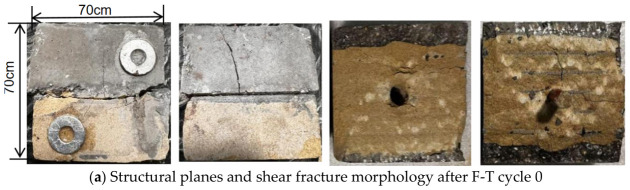

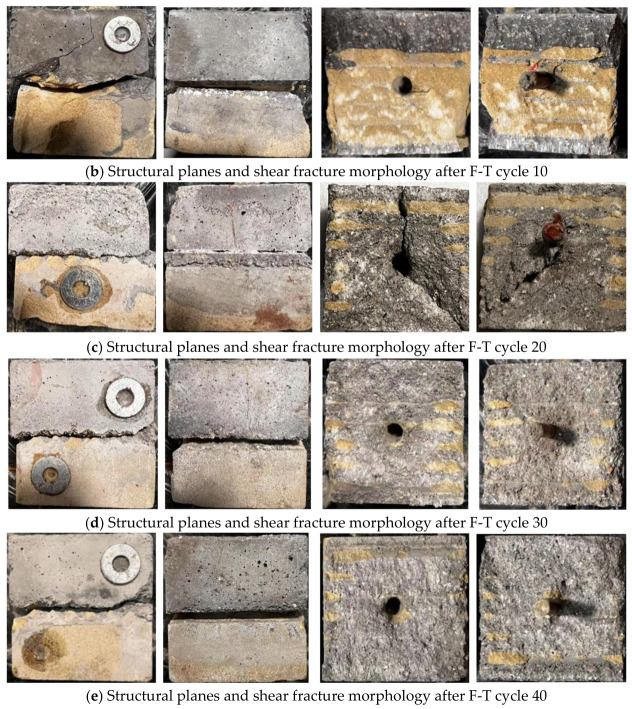
Shear failure characteristics of sandstone–concrete composite under different F-T cycle counts.

Sandstone–concrete binary specimens with a bolt depth of 25 mm and a bolt diameter of 6 mm were selected to study the shear failure mode of the binary specimens under different F-T cycles, as shown in Figure 16, which includes the structural plane failure mode and cross-sectional failure mode of the binary specimens. For specimens that have not undergone F-T cycles, the shear failure form is characterized by cracks primarily concentrated at joint surfaces, with short and straight micro-cracks forming locally at the surfaces. Individual branch cracks may propagate, primarily extending toward the sandstone side, forming elongated branch cracks. Branch cracks on the concrete side propagate to a lesser extent. The shear cross-section is primarily sandstone, with joint serrations fracturing and breaking. The cross-section surface exhibits displacement scratches, with fine sandstone powder particles adhering to the scratches. After 10 F-T cycles, the cracks at the joint surface of the specimen deepened further, with cracks on the sandstone side expanding and secondary cracks forming, reducing the surface bond strength of the sandstone. Under shear stress, block peeling occurred. On the concrete side, the cracks expanded in length but remained shallow in depth. The proportion of concrete in the shear section began to increase, and the number of displacement scratches increased. After 20 F-T cycles, the sawtooth patterns on the joint surface were damaged and peeled off, and large sandstone areas at the bottom detached. As the number of F-T cycles further increased, between 30 and 40 cycles, damage at the joint surface worsened. The concrete, being a loose and porous material, suffered severe cracking. Under shear stress, concrete particles began to fall off in large quantities. The shear surface began to concentrate primarily in the concrete region, and as the number of F-T cycles increased, the concrete content at the surface gradually increased.

### 4.2. Strain Evolution Characteristics of Sandstone–Concrete Binary Specimen Joint Surfaces Under Different Freeze–Thaw Cycles

Under external loads, rocks often begin to fail along weak surfaces, i.e., regions requiring the least energy to initiate failure. F-T cycles cause damage to rock structures, altering pore distribution and influencing failure characteristics. After anchoring the binary specimen in this test, the bolts altered the stress distribution under shear loading. Furthermore, the joint surface within the binary specimen increased the complexity of the rock mass structure. Consequently, its shear failure mode may differ from that observed in conventional direct shear tests. Further analysis of the failure characteristics is required.

**Figure 17:**
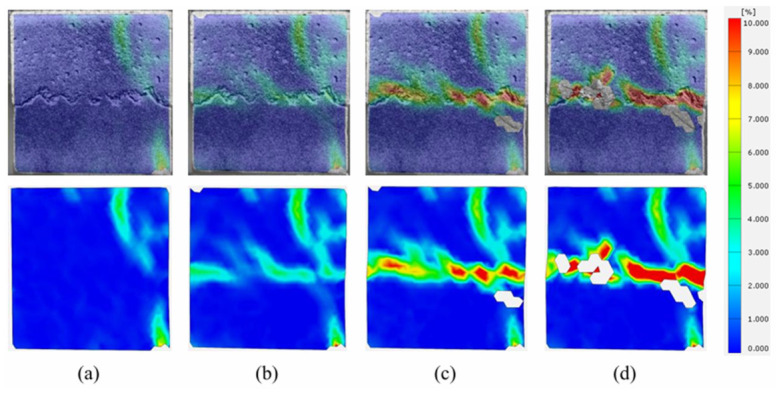
Strain evolution process at shear failure surface of binary specimens with 0 F-T cycles. (**a**) Stage I; (**b**) Stage II; (**c**) Stage III; (**d**) Stage IV.

This section analyzes the evolution of principal strains on the structural surfaces of sandstone–concrete binary specimens during shear under different F-T cycles using DIC. It investigates the crack propagation on the surface of the binary specimen after anchoring. The principal strain contour plots of the unfrozen anchored binary specimens during direct shear are shown in Figure 17a–d. At the initial contact stage, the end face of the specimen undergoes slight strain, which may be attributed to the unevenness of the specimen’s end face. As shear progresses, a strain zone extends along the right end of the joint surface on the concrete side, but it remains low in strength and does not develop into noticeable cracks. The end strain continues to extend along the sawtooth zone of the joint surface, with the strain intensity gradually increasing and developing into a strain extension zone on the concrete side. Under the influence of the end strain, cracks appear on the right side of the joint interface and expand toward the sandstone side. The sawtooth zone of the joint exhibits numerous fine, short, interlaced cracks, and small blocks of sandstone peel off at the sawtooth interface, resulting in voids in the strain map.

**Figure 18:**
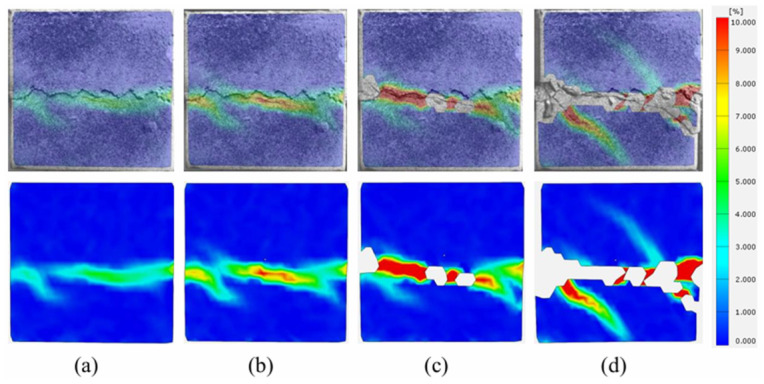
Strain evolution process at shear failure surface of binary specimens with 10 F-T cycles. (**a**) Stage I; (**b**) Stage II; (**c**) Stage III; (**d**) Stage IV.

The strain cloud diagram of the shear process of a binary specimen subjected to 10 F-T cycles is shown in Figure 18a–d. It can be observed that strain originates from the right side in the tangential loading direction. Moreover, as the shear loading progresses, it continues to develop along the joint shear plane toward the left side, giving rise to two initial cracks at the left end. After shear stress accumulates, cracking and peeling occur at the joint interface. The two initial cracks extending toward the sandstone side increase in length and depth and propagate to the specimen cross-section. After shear failure of the specimen, it can be clearly observed that both initial cracks originate from the far-left side of the joint surface and converge at a single point. One crack primarily extends along the joint surface and begins to extend downward at the midpoint of the joint surface, while the other crack primarily extends in a longitudinal direction.

**Figure 19:**
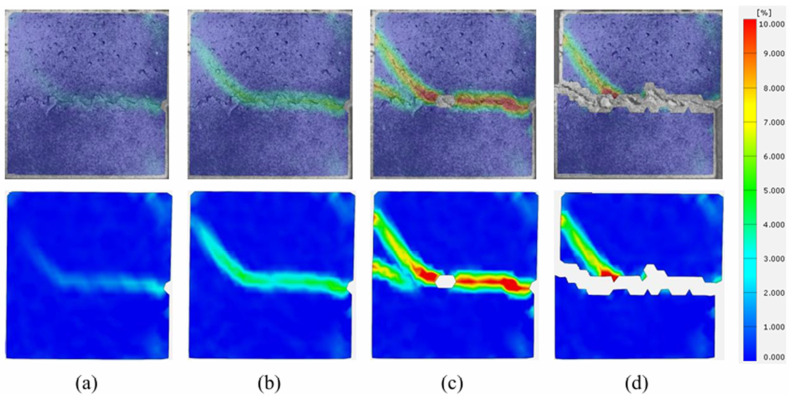
Strain evolution process at shear failure surface of binary specimens with 20 F-T cycles. (**a**) Stage I; (**b**) Stage II; (**c**) Stage III; (**d**) Stage IV.

The strain contour plots of the shear process of the binary specimen after 20 F-T cycles are shown in Figure 19a–d. Like 10 F-T cycles, strain first appears at the right end of the joint surface. As the shear displacement increases, the strain propagates along the joint surface toward the left and extends toward the concrete side at the second tooth on the left, where the first propagating crack appears. Subsequently, a second micro-crack develops at the left end of the joint surface, extending toward the concrete side, with a horizontal inclination angle greater than that of the first extended crack.

**Figure 20:**
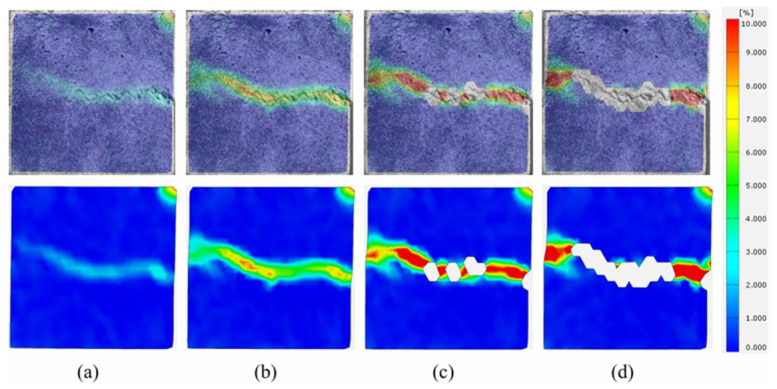
Strain evolution process at shear failure surface of binary specimens with 30 F-T cycles. (**a**) Stage I; (**b**) Stage II; (**c**) Stage III; (**d**) Stage IV.

**Figure 21:**
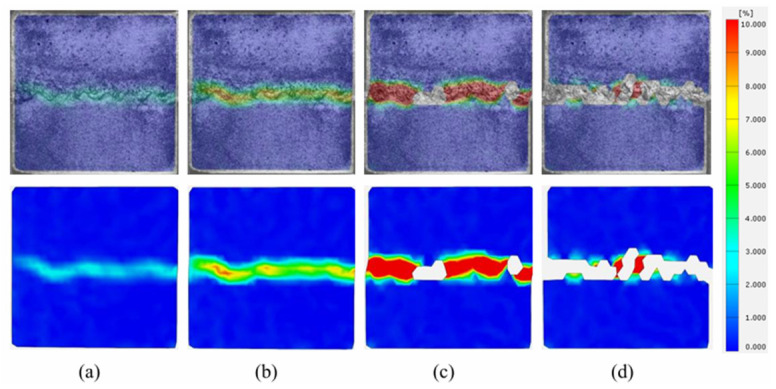
Strain evolution process at shear failure surface of binary specimens with 40 F-T cycles. (**a**) Stage I; (**b**) Stage II; (**c**) Stage III; (**d**) Stage IV.

The strain contour plots of the shear process of the binary specimen after 30 F-T cycles and 40 F-T cycles are shown in Figure 20a–d and Figure 21a–d, respectively. Comparing the two sets of figures reveals that under high F-T cycles, the strain evolution characteristics of the shear process of the anchored binary specimen are similar. The initial strain of the specimens occurs at the joint surface. As shear loading progressed, the strain spread along the joint surface toward the left, with the strain intensity gradually increasing. At 30 F-T cycles, as the strain expanded along the joint surface toward the left, it also expanded toward the concrete side of the joint surface. After the main crack formed at the joint surface, it gradually developed into a crack on the concrete side. The crack had a large inclination angle and short length, altering the expansion path of the main crack, with no other main cracks appearing. After 40 F-T cycles, no crack expansion was observed, with extensive rock detachment at the joint surface being the primary failure manifestation. This is due to the accumulation of F-T damage, which reduces the bond strength between surfaces and between surfaces and bolts. It may also be due to repeated thermal expansion of the anchoring agent during the later stages of freeze–thaw cycles, leading to a loss of bonding properties. At this stage, the primary failures are surface debonding and interface shear slippage.

## 5. Conclusions

This study is based on the research background of sprayed anchorage support for engineering slopes in cold regions, with the main rock of a particular rock slope—yellow sandstone—as the primary research object. This study investigates the mechanical properties, AE characteristics, shear failure patterns, and crack propagation of a binary specimen under different F-T cycles and bolt depths. The following conclusions are drawn:

(1) The mass damage and P-wave velocity damage of the specimens are negatively correlated with the number of F-T cycles. P-wave velocity damage is more sensitive to the number of F-T cycles than mass damage. During the early stages of F-T cycles, the moisture content increases slowly but accelerates rapidly in the later stages. F-T damage exhibits a phased characteristic, with micropore development predominating in the early stages and damage accumulation in the later stages, driven by enhanced pore connectivity.
(2) Based on NMR experiments, it was found that as the number of F-T cycles increases, the total porosity of sandstone and concrete increases, and the proportions and numbers of different pore sizes undergo complex changes, including transformations among small, medium, and large pores as well as microcracks. In the later stages of F-T cycles, the transformation from large pores to micro-cracks predominates, and surface spalling occurs. Liu, Manman, et al. [35]’s research suggests that freeze–thaw cycles have a dual effect on the micropores in sandstone: on the one hand, they cause existing micropores to expand into mesopores and macropores, resulting in a decrease in the number of micropores; on the other hand, they continuously generate new micropores within the rock matrix, ultimately leading to an initial increase followed by a decrease in the statistical frequency of micropores. This conclusion is highly similar to that presented in this paper.
(3) Anchorage depth influences the mechanical properties and F-T damage characteristics of the composite material. The peak shear strength of the composite material increases with increasing anchorage depth. As the anchorage depth increases from 0 mm to 35 mm, penetrating the surface, the composite material’s peak shear strength undergoes a phase of slow growth, rapid growth, and growth decline. The most significant increase in peak shear strength occurs between 15 mm and 25 mm anchorage depth.
(4) Based on AE characteristics, it was found that the AE ringing counts of binary system specimens under different freeze–thaw cycles showed a trend of first decreasing and then increasing during the initial freeze–thaw stage, with cumulative ringing counts first increasing, then decreasing, and then recovering. During the initial stage of F-T cycles, the proportion of shear cracks in the binary specimens first decreases and then increases in the later stage. However, tensile failure remains the dominant failure mode. Shear failure in the composite specimens occurs frequently during the initial stage of F-T cycles, with higher F-T cycles primarily characterized by surface peeling and interface slippage.
(5) An analysis of the AE characteristics and strain evolution of the binary specimen at different anchorage depths revealed that at shallow anchorage (15 mm), cracks propagate along the stress concentration zone at the bolt tip, resulting in the highest peak ring count and the lowest cumulative AE ring count. At medium anchorage depth (25 mm), tensile failure dominates, with the highest cumulative AE ring count; at deep anchorage (35 mm), the bolt induces longitudinal cracking along the bolt direction.

## Data Availability

The raw data supporting the conclusions of this article will be made available by the authors on request.
